# Double-chambered left ventricle with advanced biventricular failure: a case report

**DOI:** 10.3389/fcvm.2026.1895634

**Published:** 2026-08-27

**Authors:** Guiping Peng, Xiang Wang, Cailing Chen, Miao Dai

**Affiliations:** 1 Department of Ultrasonic and Jiujiang City Key Laboratory of Cell Therapy, Jiujiang NO. 1 People’s Hospital, Jiujiang, Jiangxi, China; 2 Department of Cardiovascular and Jiujiang City Key Laboratory of Cell Therapy, Jiujiang NO. 1 People’s Hospital, Jiujiang, Jiangxi, China; 3 Department of Anesthesiology, Jiujiang Sixth People’s Hospital, Jiujiang, Jiangxi, China; 4 Department of Geriatrics and Jiujiang City Key Laboratory of Cell Therapy, Jiujiang NO. 1 People’s Hospital, Jiujiang, Jiangxi, China; 5 Chronic Disease Management Center, Jiujiang NO. 1 People’s Hospital, Jiujiang, Jiangxi, China

**Keywords:** double-chambered left ventricle, high-burden ventricular ectopy, left ventricular non-compaction, mitral regurgitation, refractory heart failure

## Abstract

**Background:**

Double-chambered left ventricle (DCLV) is an extremely rare congenital anomaly characterized by an aberrant muscular or fibromuscular septum dividing the left ventricular cavity. Most cases present in childhood or adolescence. Adult presentations are exceptional, particularly when complicated by severe valvular dysfunction and advanced heart failure.

**Case summary:**

A 50-year-old male with longstanding atrial fibrillation presented with one month of exertional chest tightness and progressive dyspnea, including paroxysmal nocturnal dyspnea. Physical examination revealed a displaced apex and a grade 5/6 holosystolic murmur consistent with severe mitral regurgitation. Electrocardiography showed permanent atrial fibrillation with an extremely high premature ventricular complex burden (52.9%). Transthoracic echocardiography was diagnostic, demonstrating a hypertrophied muscular septum dividing the mid-to-distal left ventricle into a basal and an apical chamber (peak gradient 7 mmHg), severe mitral regurgitation due to restrictive leaflet closure, left ventricular non-compaction, and severe biventricular systolic dysfunction (left ventricular ejection fraction 29.4%). Despite optimization of guideline-directed medical therapy for heart failure with reduced ejection fraction, the patient's symptoms and functional capacity showed no improvement over one month of follow-up. Given the surgically complex anatomy, diffuse myocardial disease, and refractory biventricular failure, the multidisciplinary heart team recommended referral for orthotopic heart transplantation evaluation.

**Conclusion:**

This case highlights that double-chambered left ventricle (DCLV), though typically a pediatric diagnosis, can rarely present in adulthood with severe mitral regurgitation and refractory heart failure, adding to the limited reports of this presentation. In such cases where structural repair is not feasible due to complex anatomy and low intraventricular gradient, heart transplantation evaluation may represent the most appropriate therapeutic strategy, although the long-term response to medical therapy remains to be determined.

## Introduction

Double-chambered left ventricle (DCLV) is an exceedingly rare congenital cardiac malformation characterized by division of the left ventricular cavity into two distinct chambers by an abnormal septum, muscle bundle, fibromuscular ridge, or prominent trabeculations (1). The exact prevalence remains uncertain, with estimates ranging from 0.04% to 0.42% in the general population (2). Unlike its right-sided counterpart-double-chambered right ventricle, which is frequently associated with other congenital anomalies such as tetralogy of Fallot and ventricular septal defects-DCLV is typically an isolated finding and is far less commonly encountered in clinical practice (3).

The clinical spectrum of DCLV is remarkably heterogeneous. Many reported adult cases have been asymptomatic or minimally symptomatic, discovered incidentally during imaging for unrelated conditions (3). In a recent case report by Pomiato et al. (4), a 15-year-old girl with DCLV was initially misdiagnosed as having left ventricular noncompaction; cardiac magnetic resonance (CMR) was pivotal in establishing the correct diagnosis and guiding conservative management. Similarly, Tian et al. (2023) described an asymptomatic 33-year-old woman in whom contrast-enhanced echocardiography was necessary to confirm DCLV and differentiate it from ventricular septal defect (5). These cases underscore that when DCLV presents without hemodynamically significant obstruction or ventricular dysfunction, a conservative approach is generally favored (6).

However, the natural history and prognostic implications of DCLV when it coexists with severe systolic dysfunction, valvular regurgitation, and arrhythmic burden remain poorly characterized. Saadia S (2023) conducted a comparative analysis of pediatric and adult DCLV cases, noting that symptomatic presentations in adults are rare and typically associated with outflow tract obstruction or concomitant congenital lesions (2). Surgical intervention-most commonly resection of the obstructing septum or fibrous ring-has been described in select cases with high intraventricular gradients (7). Dogan et al. (7) reported successful surgical correction in a 13-year-old girl with obstructive DCLV, and a more recent report documented resection of a fibrous ring causing mid-ventricular stenosis in an adult patient, with favorable postoperative hemodynamic results. Notably, all previously reported surgical cases have featured significant pressure gradients across the septal partition, which provided a clear hemodynamic rationale for intervention.

Critically, the coexistence of DCLV with left ventricular non-compaction (LVNC) has been documented in only a handful of cases, invariably in pediatric populations. A landmark 2024 report described an 8-month-old infant with combined DCLV and extensive LVNC who developed refractory heart failure requiring left ventricular assist device support as a bridge to transplantation (1). Intraoperative endoscopy and histopathology confirmed the morphological overlap between these two entities, highlighting the diagnostic challenges posed by severe ventricular dilation and dysfunction. While DCLV coexisting with LVNC has been predominantly documented in pediatric populations, rare adult cases have also been reported, such as the case described by Correia et al. (2013) in a 57-year-old woman, highlighting that this association can occasionally present in adulthood (1).

Despite these important contributions, several significant gaps persist in our understanding of DCLV. First, the vast majority of reported cases have been asymptomatic or mildly symptomatic with preserved ventricular function; consequently, the management of DCLV presenting with advanced biventricular failure remains undefined. Second, previous studies have focused almost exclusively on the anatomical diagnosis and hemodynamic assessment of the intraventricular obstruction, with limited attention to the arrhythmic consequences of this structural abnormality (4). Third, while surgical resection has been advocated for obstructive DCLV with high gradients (7), there are no established management algorithms for patients in whom the intraventricular gradient is low but the myocardium is diffusely diseased, non-compacted, and functionally compromised.

We herein report a unique case of DCLV in a 50-year-old male who presented with refractory heart failure, severe biventricular dysfunction, and an extraordinarily high burden of ventricular ectopy in the context of only a minimal intraventricular pressure gradient. This case challenges the prevailing assumption that adult DCLV follows a benign trajectory and highlights the complexity of management when conventional surgical options are precluded by diffuse myopathic disease. To our knowledge, this case represents a rare adult presentation of DCLV, uniquely complicated by an extraordinarily high burden of ventricular ectopy and drug-refractory biventricular failure, which ultimately necessitated evaluation for orthotopic heart transplantation.

## Case presentation

A 50-year-old male was admitted to our cardiology department with a chief complaint of substernal chest tightness and progressive shortness of breath for one month. Symptoms were exertional and had recently progressed to paroxysmal nocturnal dyspnea requiring two pillows to sleep. His past medical history was significant for long-standing permanent atrial fibrillation, for which he was chronically anticoagulated with rivaroxaban 15 mg once daily. There was no prior history of congenital heart disease diagnosis, cardiac surgery, or systemic hypertension.

On admission, physical exam showed inferolateral displacement of the cardiac apex. Pulse was 72 bpm and irregularly irregular. Heart sounds included variable S1 intensity and a grade 5/6 holosystolic, blowing murmur at the apex radiating to the left axilla. No pericardial rub, other valvular murmurs, abdominal tenderness, peripheral edema, or jugular venous distension were noted.

**Figure 1:**
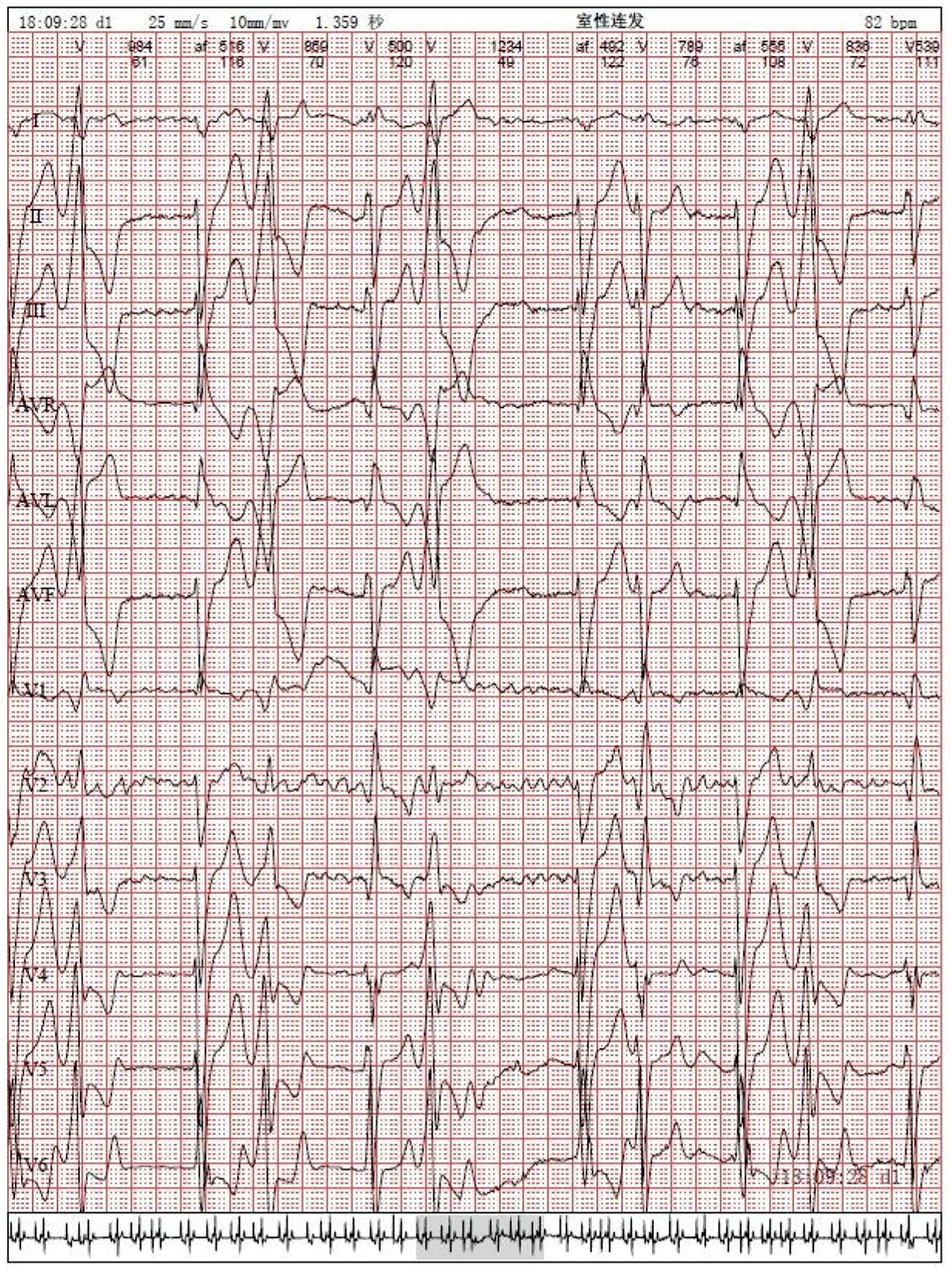
Representative 24-hour holter monitoring strip showing atrial fibrillation with frequent premature ventricular complexes and ventricular couplets. Poor R-wave progression is observed across the precordial leads (V1–V4).

**Figure 2:**
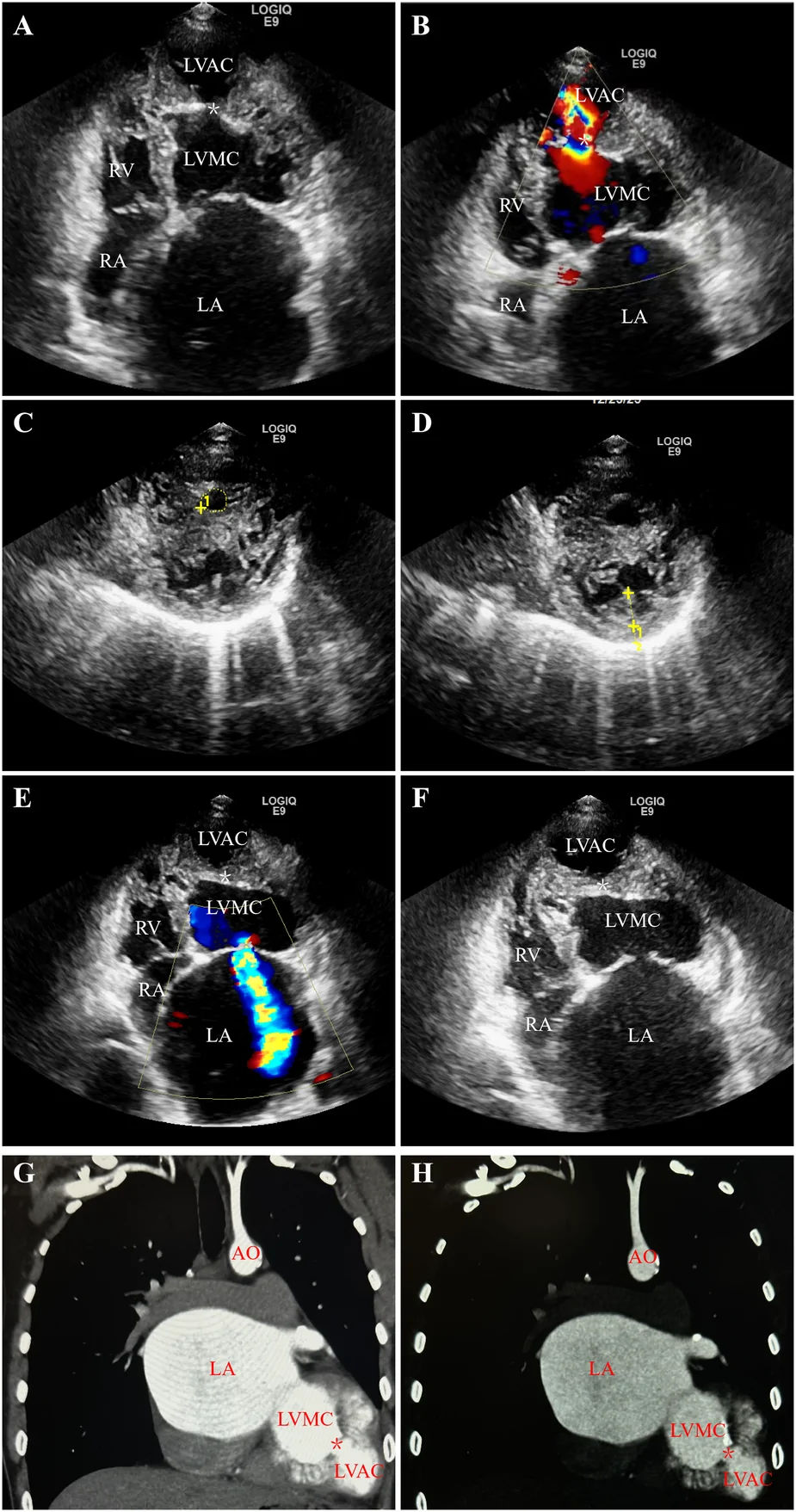
A 50-year-old male with one month of substernal chest tightness and progressive shortness of breath. **(A)** Echocardiography (Apical four-chamber view): Enlarged left heart is observed. The mid-to-lower segments of the left ventricular wall show thickening and calcification, dividing the left ventricular cavity into two chambers - one near the apex and the other adjacent to the mitral valve. **(B)** Echocardiography (Four-chamber view, Color Doppler flow imaging): Bidirectional blood flow signals between the two left ventricular chambers are visualized. **(C)** Parasternal short-axis view, sagittal ultrasound image of the intraventricular septation in the left ventricle. The measurement indicates the area of the communication opening between the two chambers. **(D)** Parasternal short-axis view. The caliper measurement indicates the thickness of the non-compacted and compacted myocardium of the left ventricular posterior wall. **(E)** Apical four-chamber view showing mitral regurgitation jet. **(F)** Apical four-chamber view, long-axis ultrasound image of the intraventricular septation in the left ventricle. **(G)** Aortic CTA (Arterial phase, original images): Enlarged left heart and double-chambered left ventricle. **(H)** Aortic CTA (Arterial phase, with adjusted grayscale): Enlarged left heart and double-chambered left ventricle. After grayscale adjustment, focal myocardial thickening with calcification is observed in the mid-portion of the left ventricle. RA, Right atrium; LA, Left atrium; RV, Right ventricle; LV, Left ventricle; LVMC, Left ventricle main cavity; LVAC, Left ventricle accessory cavity; Ao, aorta; *Muscular septum within the mid-to-lower left ventricular cavity.

High-sensitivity troponin was negative. Renal function was preserved (creatinine 75 μmol/L). Liver function tests were within normal limits (ALT 15.4 U/L, AST 15.1 U/L), as were serum electrolytes (sodium 139.7 mmol/L, potassium 4.1 mmol/L) and thyroid function (TSH 1.6 mIU/L). A comprehensive metabolic panel and thyroid profile effectively ruled out reversible metabolic, hepatic, or endocrine causes of the arrhythmia and cardiomyopathy. The clinical timeline is summarized in Table 1. On admission, N-terminal pro-B-type natriuretic peptide (NT-proBNP) was markedly elevated at 1,374 pg/mL (reference <125 pg/mL). A second sample, drawn one week later, showed an NT-proBNP of 1,580 pg/mL. Admission ECG revealed atrial fibrillation with intermittent rapid ventricular response (up to 138 bpm on Holter) and intraventricular conduction delay. The 24-hour Holter monitor documented 56,304 premature ventricular complexes (PVCs), accounting for 52.9% of total heartbeats, along with 5,877 runs of ventricular couplets and 3,806 episodes of bigeminy. A representative 24-hour Holter monitoring strip demonstrating this morphology is included as Figure 1. No sustained ventricular tachycardia was observed, but the ectopic burden was extremely high. We acknowledge that a PVC burden of this magnitude exceeds the established threshold (>24%) associated with PVC-induced cardiomyopathy. However, formal electrophysiological mapping and ablation evaluation were deferred given the patient's clinical instability and the diffuse structural disease. Transthoracic echocardiography (TTE) revealed the following (Figures 2A,B): Two-dimensional imaging showed severe left atrial dilatation (transverse diameter 8.8 cm) and left ventricular dilatation (transverse 6.9 cm, longitudinal 8.6 cm). A thick, hypertrophied muscular septum traversed the mid-to-distal left ventricular cavity, dividing it into a basal chamber and an apical chamber, with a 1.4 cm defect present in this septum. Parasternal short-axis view, sagittal ultrasound image of the intraventricular septum in the left ventricle. The area encircled represents the communication defect between the two chambers, measured at 1.09 cm^2^ (Figure 2C). Left ventricular non-compacted myocardium was noted in the lateral wall (ratio 1.6 cm non-compacted/0.5 cm compacted) (Figure 2D). On Doppler imaging, continuous-wave Doppler across the intraventricular communication revealed a peak gradient of only 7 mmHg. Severe mitral regurgitation was present due to restrictive leaflet closure (a 0.2 cm coaptation gap) and annulo-ventricular dilation. Quantitative assessment by the proximal isovelocity surface area (PISA) method yielded an effective regurgitant orifice area (EROA) of 0.52 cm^2^ and a regurgitant volume of 68 mL/beat, with a vena contracta width of 0.7 cm-all meeting the guideline-defined criteria for severe MR (EROA ≥0.40 cm^2^, regurgitant volume ≥60 mL, vena contracta ≥0.7 cm) (Figures 2C,D). right ventricular size (basal diameter 4.1 cm, mid-cavity 3.3 cm, longitudinal 7.2 cm) with impaired function (TAPSE 1.1 cm, RVFAC 31%, S’ 8.2 cm/s). Systolic function was severely reduced in both ventricles: left ventricular ejection fraction (LVEF) by Simpson's biplane method was 29.4%, and right ventricular function was also impaired (TAPSE 1.1 cm, RVFAC 31%). In conclusion, the TTE findings were consistent with left ventricular double-chambered heart, severe mitral regurgitation, severe left ventricular dysfunction, moderate right ventricular dysfunction, mild pulmonary hypertension (estimated PASP 43 mmHg), and a small pericardial effusion. Cardiac magnetic resonance imaging was considered the gold standard for definitive tissue characterization; however, it was not feasible in this case due to patient-related factors, including permanent atrial fibrillation with an extremely high premature ventricular complex burden (52.9%) and clinical instability (NYHA class III-IV) that precluded prolonged supine scanning. Left ventricular non-compacted myocardium was diagnosed using the Jenni criteria, which require: (1) a two-layered myocardial structure with a prominent non-compacted endocardial layer and a thin compacted epicardial layer; (2) a non-compacted/compacted (NC/C) ratio >2.0 measured in end-diastole; and (3) color Doppler evidence of deep intertrabecular recesses perfused from the ventricular cavity. The NC/C ratio was measured in the lateral wall on apical four-chamber view, at the end-diastolic frame immediately preceding mitral valve closure, using the maximal thickness of the non-compacted layer (1.6 cm) divided by the compacted layer (0.5 cm), yielding a ratio of 3.2, exceeding the diagnostic threshold. Color Doppler confirmed blood flow within the deep intertrabecular recesses. The other LVNC diagnostic criteria (Petersen: NC/C ratio >2.3 on short-axis CMR; Jacquier: trabeculated mass >20% of total LV mass on CMR) are CMR-based and were not applicable in this case due to patient-related contraindications to cardiac magnetic resonance imaging.

**Table 1:** Clinical timeline of the case.

Time point	Clinical events, diagnostic findings & interventions
>2 years before admission	- Gradual onset of chronic exertional dyspnea and palpitations. - Treated intermittently with low-dose beta-blockers and digoxin without specialist cardiology follow-up.
1 month before admission	- Acute exacerbation: Progressive shortness of breath, substernal chest tightness, and development of paroxysmal nocturnal dyspnea. - Patient seeks care, leading to admission.
Day of admission (Day 0)	- Physical Exam: Displaced apex, grade 5/6 holosystolic murmur. - ECG: Atrial fibrillation with rapid ventricular response and intermittent intraventricular conduction delay. - Lab: NT-proBNP markedly elevated at 1,374 pg/mL.
Day 1–4 (Inpatient Diagnostic Workup)	- 24-hour Holter Monitor: 56,304 PVCs (52.9% burden), 5,877 runs of ventricular couplets, 3,806 episodes of bigeminy. - Echocardiography (TTE): Diagnostic for DCLV, severe mitral regurgitation (EROA 0.52 cm^2^), severe biventricular dysfunction (LVEF 29.4%), and LVNC (NC/C ratio 3.2:1). - aortic CTA: Confirmed LV enlargement and showed focal mid-ventricular calcification.
Day 2–14 (Therapeutic Initiation & Observation)	- Initiation of guideline-directed medical therapy (GDMT) for HFrEF: Sacubitril/valsartan, dapagliflozin, and metoprolol succinate. - Continued rivaroxaban for stroke prevention. - Observation: No clinical improvement in symptoms (dyspnea, fatigue) or functional capacity over a 2-week period of inpatient observation.
Day 15 (Multidisciplinary Decision)	- Heart Team Consensus: The patient's complex anatomy (DCLV + diffuse LVNC), low intraventricular gradient (making resection unhelpful), severe functional MR, and refractory biventricular failure made surgical correction unfeasible. - Decision: Patient was formally referred for orthotopic heart transplantation evaluation.
At Discharge and Ongoing	- Discharged on optimized GDMT, awaiting a suitable donor heart. - Ongoing Plan: Outpatient pre-transplantation evaluation, including INTERMACS profile, cardiopulmonary exercise testing (peak VO₂), right heart catheterization (PVR measurement), and transplantation committee review.

ECG, electrocardiography; NT-proBNP, N-terminal pro-B-type natriuretic peptide; PVCs, premature ventricular complexes; TTE, transthoracic echocardiography; EROA, effective regurgitant orifice area; LVEF, left ventricular ejection fraction; LVNC, left ventricular non-compaction; NC/C, non-compacted/compacted; CTA, computed tomography angiography; HFrEF, heart failure with reduced ejection fraction; DCLV, double-chambered left ventricle; MR, mitral regurgitation; GDMT, guideline-directed medical therapy; PVR, pulmonary vascular resistance.

Aortic computed tomography angiography (aortic CTA) demonstrated a markedly enlarged left ventricle with focal myocardial thickening and calcification in the mid-ventricular segment (Figures 2C,D). A small pericardial effusion was noted, and there was no evidence of aortic dissection or coronary artery stenosis beyond mild atherosclerotic plaques (Figures 2C,D). Given the absence of ischemic symptoms and negative high-sensitivity troponin, invasive coronary angiography was not pursued.

The final diagnosis was: (1) Chronic heart failure with acute exacerbation (NYHA functional class III-IV) secondary to left ventricular double-chambered heart; (2) Severe mitral regurgitation; (3) Permanent atrial fibrillation with high-density ventricular ectopy (52.9% PVC burden); (4) Biventricular failure with systolic dysfunction (LVEF 29.4%).

It is important to note that the patient had a history of chronic exertional dyspnea and palpitations for over two years prior to admission, for which he had been treated with low-dose beta-blockers and digoxin intermittently without specialist follow-up. The one-month acute deterioration represented decompensation of a long-standing, structurally advanced disease. The patient was started on guideline-directed medical therapy for heart failure with reduced ejection fraction (HFrEF), including sacubitril/valsartan (target 97/103 mg twice daily), dapagliflozin 10 mg daily, and metoprolol succinate (titrated to 100 mg daily) for rate control. Rivaroxaban was continued for stroke prevention in atrial fibrillation. Despite this optimal regimen, his symptoms (dyspnea on minimal exertion, fatigue) and functional capacity did not improve over a two-week period of inpatient observation. The structural abnormality (DCLV) was deemed surgically complex due to the diffusely diseased myocardium, non-compaction, and the presence of only a low-pressure gradient across the septum (making simple resection of an obstructing septum less beneficial and potentially hazardous). Given the persistence of advanced heart failure symptoms despite initial medical optimization, the high arrhythmic burden, and the severity of mitral regurgitation, a multidisciplinary heart team consensus was reached to refer the patient for orthotopic heart transplantation evaluation. He was discharged home on optimal medical therapy and is currently undergoing outpatient pre-transplantation workup, including formal assessments such as INTERMACS profile, cardiopulmonary exercise testing with peak VO₂, right heart catheterization, pulmonary vascular resistance measurement, and transplantation committee review. The final listing decision will be made upon completion of these evaluations. The decision to list was based on multidisciplinary consensus considering the irreversible structural substrate and refractory biventricular failure.

At one-month outpatient follow-up, the patient remained in NYHA class III despite optimal medical therapy, with NT-proBNP rising to 1,820 pg/mL. No improvement in LVEF (29.8%) or right ventricular function was observed on repeat echocardiography. These objective findings confirm persistent advanced biventricular failure over short-term follow-up and support the decision to pursue heart transplantation evaluation, though longer-term reverse remodeling cannot be entirely excluded.

## Discussion

Double-chambered left ventricle (DCLV), also referred to as cor triventriculare sinistrum, is an exceedingly rare congenital anomaly in which an abnormal muscular septum divides the left ventricular cavity into two distinct chambers (2). Our case is notable for presenting in a 50-year-old male with advanced biventricular failure, severe mitral regurgitation, and a remarkably high ventricular ectopic burden-features that, in aggregate, represent a far more malignant phenotype than is typically reported.

The anatomical classification of DCLV remains heterogeneous. Recently, Bao et al. proposed a simplified morphology-based classification (superior-inferior vs. parallel), noting that the former is more readily diagnosed by echocardiography (8). In our patient, a hypertrophied muscular septum divided the mid-to-distal left ventricle into basal and apical chambers via a 1.4-cm septal defect-consistent with the superior–inferior type, which carries higher rates of associated ventricular septal defects, mitral regurgitation, and LVNC (8). Indeed, Bao et al. reported that 9 pediatric patients with superior-inferior DCLV frequently exhibited associated structural abnormalities, including LVNC in one case (8). The presence of LVNC in our patient (non-compacted/compacted ratio of 1.6 cm/0.5 cm in the lateral wall) further complicates the anatomical substrate. Novo et al. (9) previously described a case in which LVNC mimicked DCLV on imaging, and Correia et al. (10) reported a case of true coexistence in a 57-year-old woman, highlighting the diagnostic overlap and the rare but established occurrence of these two entities in adults.

However, our patient demonstrated echocardiographic findings consistent with both conditions: a discrete muscular septum dividing the LV cavity (consistent with DCLV) alongside prominent trabeculations and deep intertrabecular recesses fulfilling Jenni criteria for LVNC (9). The pathophysiology of adult DCLV is poorly understood and appears influenced by intraventricular obstruction severity, septal integrity, and associated lesion burden (2). Our continuous-wave Doppler showed a peak gradient of only 7 mmHg across the intraventricular communication-far below the 110-mmHg surgical threshold. This contrasts with published reports in younger symptomatic patients, where successful resection with patch reconstruction was performed (2).

Diagnosing DCLV in adults is challenging and requires multimodality imaging. Initial transthoracic echocardiography is frequently misdiagnosed as diverticulum, aneurysm, ventricular septal defect, or LVNC (8). TTE diagnosed a hypertrophied muscular septum dividing the mid-to-distal LV into basal high-pressure and apical low-pressure chambers (with a 1.4 cm defect). CTA added anatomic details-mid-ventricular thickening and calcification-and excluded CAD. Cardiac magnetic resonance imaging, considered the gold standard for tissue characterization, was not feasible due to permanent atrial fibrillation with an extremely high PVC burden (52.9%) and clinical instability. The coexistence of findings consistent with DCLV (a discrete muscular septum dividing the LV cavity) and LVNC (NC/C ratio 3.2 in the lateral wall, with color Doppler evidence of deep intertrabecular recesses) underscores the diagnostic overlap between these rare entities in adults.

In this patient, the peak intraventricular gradient was only 7 mmHg-far below hemodynamically significant obstruction-so mechanical blockage was not the main cause of heart failure. Rather, the phenotype arises from four interacting mechanisms: (i) LVNC-related non-compaction cardiomyopathy; (ii) PVC-induced cardiomyopathy (52.9% burden, well above the >24% threshold for reversible dysfunction); (iii) tachycardia-mediated remodeling from chronic atrial fibrillation; and (iv) severe functional mitral regurgitation (MR) driving volume overload. The severe MR resulted from restrictive leaflet closure (0.2 cm coaptation gap) and annulo-ventricular dilation due to LV geometric distortion, not primary leaflet disease-creating a vicious cycle of volume overload, massive left atrial dilatation (8.8 cm), and biventricular systolic decline. The high ventricular ectopic burden likely served as both marker and contributor to progression, though formal mapping/ablation was deferred given the diffuse structural disease and clinical instability.

The patient was started on guideline-directed medical therapy for HFrEF (sacubitril/valsartan, dapagliflozin, metoprolol) (11) plus rivaroxaban for AF-related stroke prevention-a strategy increasingly supported in heart disease. Other interventions were systematically ruled out: PVC ablation was deferred given multifocal morphology without a dominant focus, indicating diffuse myocardial disease rather than a treatable driver; even with 52.9% burden, fixed structural damage and severe MR limit recovery, making ablation unfavorable in this unstable patient (NYHA III–IV). Rhythm control for AF was not prioritized due to permanent AF (>2 years), massive left atrial dilation (8.8 cm), and extensive fibrosis, with adequate rate control (72–85 bpm) and anticoagulation already in place. ICD/wearable defibrillator implantation was deferred as the patient is under transplant evaluation-the only definitive therapy-and a device could complicate candidacy. CRT was not indicated (narrow QRS, 98 ms). Mitral intervention/TEER was deemed unlikely to offer durable benefit given purely functional MR without primary leaflet disease, with high failure risk and prohibitive surgical risk in biventricular failure with diffuse LVNC. MCS was discussed but not pursued, as the patient remains hemodynamically stable (no inotrope dependence, no end-organ dysfunction) without transplant contraindications, offering no advantage over medical therapy during evaluation. Ultimately, transplantation remains the only option addressing both the structural anomaly and diffuse biventricular failure.

While guideline-directed medical therapy typically requires months for maximal reverse remodeling, the fixed anatomical substrate-DCLV, diffuse LVNC, and severe biventricular dysfunction-suggests limited recovery potential. However, one month of follow-up is too short to confirm irreversibility. The transplantation evaluation was recommended by multidisciplinary consensus, given the complex anatomy precluding surgical repair, persistent advanced heart failure symptoms despite initial optimization, and lack of alternatives. Objective listing criteria (INTERMACS, peak VO₂, hemodynamics) were unavailable at submission but are being completed sequentially; the final decision will rely on these data. Here, “refractory” describes the clinical response during the available follow-up, not an irreversible prognosis. This contrasts with Dogan et al.'s successful surgical management of a 13-year-old girl with DCLV and a significant gradient (12). More recently, Nie et al. reported a case of DCLV with a giant thrombus managed surgically (13). However, none of these cases involved the constellation of biventricular failure, severe MR, and massive PVC burden seen in our patient. To our knowledge, this may represent one of the rare adult DCLV cases complicated by advanced biventricular failure and high ventricular ectopic burden requiring consideration of advanced heart failure therapies.

## Conclusion

This case shows that adult DCLV may present with a lethal triad of biventricular failure, severe functional MR, LVNC, and high arrhythmic burden. Absence of significant obstruction likely indicates advanced disease with exhausted compensatory mechanisms, not a benign form. In low-gradient DCLV with diffuse dysfunction and no surgical options, transplant evaluation is appropriate, though the long-term response to medical therapy is uncertain.

## Data Availability

The raw data supporting the conclusions of this article will be made available by the authors, without undue reservation.
